# Supplementary material for: Racial and ethnic differences in epigenetic aging: The National Health and Nutrition Examination Survey, 1999–2002
Source: PLoS One. 2025 Jul 16;20(7):e0327010. doi: 10.1371/journal.pone.0327010 (PMC12266396; doi:10.1371/journal.pone.0327010)
Supplement: S1 Table — Model 1 adjusts for chronological age, age-squared, gender, nativity, and blood cell composition (% lymphocytes, % monocytes, % segmented neutrophils, % eosinophils, and % basophils). Model 2 adjusts for all covariates in Model 1, plus marital status, educational attainment, poverty income ratio, and occupation. Model 3 adjusts for all covariates in Models 1 and 2, plus smoking, alcohol consumption, physical activity, and diet. Beta coefficients and 95% confidence intervals in bold are significant at the p < 0.05 level. (DOCX) [file pone.0327010.s001.docx]

| S1 Table. Linear Regression Models Examining Racial and Ethnic Differences in Epigenetic Aging with Adjustment for Blood Cell Composition, NHANES 1999-2002 (n=2,402) | | | |
| --- | --- | --- | --- |
|  | Model 1 | Model 2 | Model 3 |
|  | Beta (95% CI) | Beta (95% CI) | Beta (95% CI) |
| *DNAm Chronological Age Measures* | | | |
| Hannum |  |  |  |
| Non-Hispanic Black | Ref. | Ref. | Ref. |
| Non-Hispanic White | **1.34 (0.76, 1.91)** | **1.48 (0.81, 2.15)** | **1.46 (0.70, 2.21)** |
| Mexican American | **2.94 (1.82, 4.05)** | **2.60 (1.47, 3.73)** | **2.69 (1.57, 3.78)** |
| Other Hispanic | **1.56 (0.62, 2.50)** | **1.27 (0.31, 2.24)** | **1.29 (0.48, 2.14)** |
| Another race or ethnicity | **1.99 (0.53, 3.45)** | **1.83 (0.35, 3.30)** | **1.73 (0.25, 3.20)** |
| Horvath |  |  |  |
| Non-Hispanic Black | Ref. | Ref. | Ref. |
| Non-Hispanic White | 0.63 (-0.09, 1.36) | 0.63 (-0.13, 1.40) | 0.67 (-0.15, 1.49) |
| Mexican American | 0.48 (-0.57, 1.53) | 0.29 (-0.84, 1.42) | 0.31 (-0.80, 1.41) |
| Other Hispanic | -0.18 (-1.06, 0.70) | -0.19 (-1.19, 0.81) | -0.23 (-1.13, 0.66) |
| Other race or ethnicity | 0.34 (-1.07, 1.76) | 0.24 (-1.27, 1.76) | 0.14 (-1.38, 1.66) |
| Weidner |  |  |  |
| Non-Hispanic Black | Ref. | Ref. | Ref. |
| Non-Hispanic White | **0.95 (0.01, 1.90)** | 0.84 (-0.25, 1.93) | 0.83 (-0.43, 2.08) |
| Mexican American | 0.69 (-0.37, 1.74) | 0.58 (-0.65, 1.80) | 0.54 (-0.66, 1.75) |
| Other Hispanic | -1.30 (-3.32, 0.73) | -1.39 (-3.43, 0.66) | -1.47 (-3.58, 0.64) |
| Other race or ethnicity | -1.89 (-4.62, 0.85) | -2.07 (-4.98, 0.84) | -1.99 (-4.95, 0.96) |
| Vidal-Bralo |  |  |  |
| Non-Hispanic Black | Ref. | Ref. | Ref. |
| Non-Hispanic White | **1.04 (0.48, 1.60)** | **0.98 (0.39, 1.58)** | **0.96 (0.28, 1.64)** |
| Mexican American | 0.16 (-0.55, 0.86) | -0.05 (-0.77, 0.68) | -0.01 (-0.73, 0.70) |
| Other Hispanic | -0.31 (-1.12, 0.49) | -0.42 (-1.26, 0.41) | -0.44 (-1.26, 0.38) |
| Other race or ethnicity | -0.90 (-1.84, 0.05) | **-1.06 (-2.02, -0.10)** | -1.00 (-1.97, -0.02) |
| Lin |  |  |  |
| Non-Hispanic Black | Ref. | Ref. | Ref. |
| Non-Hispanic White | -0.24 (-1.28, 0.81) | -0.35 (-1.50, 0.80) | -0.30 (-1.52, 0.92) |
| Mexican American | -0.22 (-1.70, 1.26) | -0.47 (-2.06, 1.11) | -0.56 (-2.16, 1.03) |
| Other Hispanic | -1.36 (-3.23, 0.51) | -1.24 (-3.18, 0.69) | -1.40 (-3.27, 0.48) |
| Other race or ethnicity | -1.09 (-2.55, 0.38) | -1.16 (-2.72, 0.40) | -1.33 (-2.83, 0.17) |
| SkinBlood |  |  |  |
| Non-Hispanic Black | Ref. | Ref. | Ref. |
| Non-Hispanic White | **0.68 (0.06, 1.30)** | **0.68 (0.03, 1.33)** | **0.76 (0.05, 1.47)** |
| Mexican American | **1.11 (0.15, 2.07)** | 0.77 (-0.20, 1.73) | 0.82 (-0.14, 1.78) |
| Other Hispanic | 0.79 (-0.12, 1.71) | 0.65 (-0.33, 1.63) | 0.62 (-0.22, 1.47) |
| Other race or ethnicity | 1.17 (-0.21, 2.55) | 1.04 (-0.40, 2.47) | 0.90 (-0.51, 2.32) |
| Zhang |  |  |  |
| Non-Hispanic Black | Ref. | Ref. | Ref. |
| Non-Hispanic White | **0.53 (0.32, 0.75)** | **0.51 (0.27, 0.74)** | **0.52 (0.26, 0.78)** |
| Mexican American | **0.50 (0.13, 0.87)** | 0.39 (-0.01, 0.78) | **0.40 (0.02, 0.79)** |
| Other Hispanic | **0.49 (0.16, 0.82)** | **0.44 (0.08, 0.80)** | **0.43 (0.10, 0.75)** |
| Other race or ethnicity | 0.32 (-0.17, 0.81) | 0.23 (-0.28, 0.75) | 0.18 (-0.32, 0.68) |
| *DNAm Physiological Age Measures* | | | |
| PhenoAge |  |  |  |
| Non-Hispanic Black | Ref. | Ref. | Ref. |
| Non-Hispanic White | **-0.86 (-1.70, -0.02)** | -0.37 (-1.26, 0.51) | -0.44 (-1.32, 0.45) |
| Mexican American | 1.13 (-0.19, 2.45) | 0.70 (-0.64, 2.03) | 0.85 (-0.41, 2.11) |
| Other Hispanic | -0.20 (-1.44, 1.04) | -0.60 (-1.88, 0.68) | -0.60 (-1.90, 0.70) |
| Other race or ethnicity | 0.58 (-1.41, 2.56) | 0.53 (-1.29, 2.35) | 0.50 (-1.35, 2.34) |
| GrimAge |  |  |  |
| Non-Hispanic Black | Ref. | Ref. | Ref. |
| Non-Hispanic White | **-2.39 (-3.04, -1.74)** | **-1.47 (-2.09, -0.84)** | **-1.93 (-2.43, -1.44)** |
| Mexican American | **-1.45 (-2.57, -0.33)** | **-1.55 (-2.40, -0.69)** | **-1.41 (-2.01, -0.81)** |
| Other Hispanic | **-1.65 (-2.86, -0.44)** | **-2.03 (-3.18, -0.89)** | **-2.23 (-3.11, -1.36)** |
| Other race or ethnicity | **-1.16 (-2.28, -0.03)** | -0.72 (-1.79, 0.35) | **-1.10 (-1.83, -0.37)** |
| DunedinPoAm |  |  |  |
| Non-Hispanic Black | Ref. | Ref. | Ref. |
| Non-Hispanic White | -0.06 (-0.07, -0.04) | **-0.04 (-0.06, -0.03)** | **-0.05 (-0.06, -0.04)** |
| Mexican American | -0.02 (-0.04, -0.00) | **-0.03 (-0.04, -0.01)** | **-0.02 (-0.04, -0.01)** |
| Other Hispanic | -0.02 (-0.04, 0.00) | **-0.03 (-0.04, -0.00)** | **-0.03 (-0.04, -0.01)** |
| Other race or ethnicity | -0.01 (-0.03, 0.02) | -0.00 (-0.03, 0.02) | -0.01 (-0.03, 0.01) |
| GrimAge2 |  |  |  |
| Non-Hispanic Black | Ref. | Ref. | Ref. |
| Non-Hispanic White | **-3.38 (-4.08, -2.68)** | **-2.28 (-2.92, -1.64)** | **-2.70 (-3.23, -2.17)** |
| Mexican American | **-1.53 (-2.71, -0.35)** | **-1.69 (-2.57, -0.81)** | **-1.49 (-2.11, -0.87)** |
| Other Hispanic | **-1.62 (-2.86, -0.38)** | **-2.09 (-3.25, -0.93)** | **-2.21 (-3.13, -1.29)** |
| Other race or ethnicity | **-1.35 (-2.66, -0.04)** | -0.84 (-1.98, 0.31) | **-1.19 (-2.09, -0.33)** |
| *DNAm Biomarkers of Aging Measures* | | | |
| Yang |  |  |  |
| Non-Hispanic Black | Ref. | Ref. | Ref. |
| Non-Hispanic White | **-0.00 (-0.01, -0.00)** | **-0.00 (-0.01, -0.00)** | **-0.00 (-0.01, -0.00)** |
| Mexican American | **0.00 (0.00, 0.01)** | 0.00 (-0.00, 0.01) | 0.00 (-0.00, 0.01) |
| Other Hispanic | -0.00 (-0.01, 0.00) | -0.00 (-0.01, 0.00) | -0.00 (-0.01, 0.00) |
| Other race or ethnicity | 0.00 (-0.00, 0.01) | 0.00 (-0.00, 0.01) | -0.00 (-0.00, 0.01) |
| Telomere |  |  |  |
| Non-Hispanic Black | Ref. | Ref. | Ref. |
| Non-Hispanic White | **-0.18 (-0.21, -0.16)** | **-0.21 (-0.24, -0.18)** | **-0.19 (-0.22, -0.17)** |
| Mexican American | **-0.21 (-0.26, -0.16)** | **-0.20 (-0.24, -0.15)** | **-0.20 (-0.24, -0.15)** |
| Other Hispanic | **-0.17 (-0.24, -0.11)** | **-0.16 (-0.22, -0.10)** | **-0.16 (-0.22, -0.10)** |
| Other race or ethnicity | **-0.23 (-0.28, -0.18)** | **-0.24 (-0.29, -0.19)** | **-0.23 (-0.28, -0.18)** |
| Model 1 adjusts for chronological age, age-squared, gender, nativity, and blood cell composition (% lymphocytes, % monocytes, % segmented neutrophils, % eosinophils, and % basophils). Model 2 adjusts for all covariates in Model 1, plus marital status, educational attainment, poverty income ratio, and occupation. Model 3 adjusts for all covariates in Models 1 and 2, plus smoking, alcohol consumption, physical activity, and diet. Beta coefficients and 95% confidence intervals in bold are significant at the p<0.05 level. | | | |
