# Supplementary material for: Racial and ethnic differences in epigenetic aging: The National Health and Nutrition Examination Survey, 1999–2002
Source: PLoS One. 2025 Jul 16;20(7):e0327010. doi: 10.1371/journal.pone.0327010 (PMC12266396; doi:10.1371/journal.pone.0327010)
Supplement: Attachment — Submitted filename: RPLOSONEFINISH.docx [file pone.0327010.s002.docx]

**Manuscript Title:** Racial and Ethnic Differences in Epigenetic Aging: The National Health and Nutrition Examination Survey, 1999-2002

**Introduction**

The introduction does an admirable job of emphasizing the importance of the study by focusing on the "weathering" hypothesis, which suggests a potential link between accelerated biological aging and racial and ethnic disparities in the U.S. By grounding the argument in relevant literature, the author provides a solid theoretical framework, highlighting the ways in which psychosocial and biological exposures can influence health outcomes. Furthermore, the study is positioned as addressing a key gap in research, namely the absence of consensus on biological aging measures. This makes its purpose both timely and compelling.

Two clear objectives are outlined: investigating racial and ethnic differences in epigenetic aging and examining the role of socioeconomic and behavioral factors in attenuating these differences. The discussion of DNA methylation and its relevance to health outcomes provides a strong foundation for understanding the study's goals. However, the introduction could be enhanced by providing greater detail about the epigenetic markers used and their connection to health disparities, as well as a stronger emphasis on how the predominance of white-based epigenetic measures may limit broader applicability.

**Methodology**

The methods section is comprehensive, providing clear descriptions of the data sources, sampling strategies, and analytical approaches. The use of NHANES data, combined with robust protocols for DNA methylation analysis, enhances the study's reliability and replicability. Moreover, the inclusion of minority oversampling and adjustments for demographic and behavioral covariates ensures a thoughtful and inclusive approach to the analysis.

While the technical details are well-documented, some areas could be expanded for clarity. For instance, the rationale behind selecting specific epigenetic measures is not thoroughly explained, leaving readers to infer their relevance. Similarly, the mention of multiple imputations for missing data would benefit from additional context on its validation and potential impact on results. Lastly, a more explicit discussion of potential biases in the epigenetic measures—especially considering their development within predominantly white populations—would strengthen this section.

**Results**

The results are clearly presented, with detailed statistical summaries and thoughtful integration of tables and figures. The focus on multiple epigenetic aging measures provides a nuanced analysis of racial and ethnic differences. However, certain results that deviate from the initial hypothesis, such as instances where white participants exhibit greater epigenetic aging, could be better contextualized.

While the statistical significance of findings is well-addressed, the practical implications of some results—particularly in measures like telomeres—could be further emphasized. The authors might also consider discussing potential analytical limitations, such as biases introduced by oversampling minorities or the validation gaps in epigenetic measures for non-white populations.

**Discussion**
The discussion builds effectively on the study’s findings, linking results to the theoretical framework and broader literature. The analysis of discrepancies between expected and observed outcomes, such as the unexpected epigenetic aging patterns in white participants, is particularly commendable. This reflective approach enhances the credibility of the study.

Despite these strengths, the discussion could delve deeper into the social and biological factors behind these unexpected results. Additionally, while the study acknowledges racial health disparities, it stops short of exploring how these findings might influence public health policies or interventions. A more explicit connection between epigenetic measures and tangible health outcomes, such as morbidity or mortality rates, would also add depth and relevance.

**Conclusion**
The conclusion succinctly summarizes the study’s contributions, reaffirming its relevance to public health and epigenetic research. By emphasizing the need for validation of epigenetic measures in diverse populations, it sets a clear direction for future studies. However, the practical applications of the findings—particularly for public health strategies—are not fully explored. A more direct discussion of how this research could inform policies to address racial health disparities would significantly enhance its impact.

Recommendations
To refine the manuscript and ensure greater engagement:

Connect Findings to Broader Implications: Strengthen the link between unexpected results and theoretical or practical explanations, particularly in the discussion.

Expand the Scope of Policy Relevance: Emphasize how the findings could inform interventions or monitoring of racial health disparities.

Address Methodological Gaps: Provide more justification for the selection of epigenetic measures and a deeper discussion of their limitations in racially diverse populations.
